# Axillary recurrences after sentinel node (SLN) biopsy without complete axillary dissection in breast cancer patients

**DOI:** 10.1038/sj.bjc.6602430

**Published:** 2005-03-01

**Authors:** P Kristen, R Lorenz, M Gross, A Beck, M Sütterlin, C Reiners, J Dietl

**Affiliations:** 1 Department of Obstetrics and Gynecology, Julius-Maximilians-Universität Würzburg, Germany; 2 Department of Nuclear Medicine; Julius-Maximilians-Universität Würzburg, Germany

**Sir**,

We read with interest the paper by Reitsamer *et al* (2004) regarding SLN biopsy without axillary node dissection in a group of 200 patients proven sentinel node negative based on histology (frozen and paraffin-embedded sections) and immunhistochemistry. After a mean follow-up of 36 months (22–56 months), no axillary recurrence has been found so far.

In our own collective, we surveyed 120 consecutive patients with the sentinel technique. We started at the end of 2001, after completion of a learning phase with 54 patients. We used the same combination of radionuclide and blue dye labelling as described in the paper by Reitsamer *et al* (2004). In these patients, the sentinel node was detectable in 93.3% (*n*=112). A total of 25 patients (22.3%) had a positive sentinel node (proven by histology or immunhistochemistry), which led to primary or secondary complete axillary dissection. After a mean follow-up of 13.5 months (2–27 months), we have found up until this day one case of axillary recurrence. In contrast to the paper of Reitsamer *et al* (2004), the rate of positive sentinel nodes in our experience was much lower (22.3 *vs* 39.0%). A possible reason for this could be due to the fact that we discriminate the patients for the sentinel node technique not only by clinically negative axillae but also by sonographic negative axillae. Patients with sonographic conspicuous nodes are excluded from the sentinel node biopsy.

In our opinion, the sentinel node technique is well established in clinical settings. The data of different large studies show a sensitivity of about 92% for the sentinel node technique (Kuehn *et al*, 2004). This means that approximately eight out of 100 patients could be mis-staged, which might lead to an inadequate adjuvant treatment and increased regional recurrence. On the other hand, a much larger number of patients will be spared the morbidity of axillary dissection, with risk of lymphoedema, seroma and paraesthesia of the ipsilateral arm. Ideally, we should wait for the results of large controlled trials, such as NSABP-32, to calculate the estimated survival of patients, but these trials are not due to be completed until 2006 (Veronesi *et al*, 2003; Beechey-Newman 2004). From other studies, we know that local recurrence does not seem to influence the survival of the patients (Fisher *et al*, 2002); the therapeutic use of complete axillary dissection is still controversial (Recht and Houlihan, 1995) and today most of our patients receive adjuvant therapy after primary surgery. So, with regard to the large number of data, we can no longer accept the assessment by Reitsamer *et al* (2004) that ‘axillary lymph node dissection should not be abandoned as standard care for SLN-negative patients’. If nothing else, our informed patients expect us to know, to be trained in and to apply this technique in our routine treatment.
